# Choreographies of co-modification: instrumentizing cod for immunology and the economy

**DOI:** 10.1007/s40656-025-00677-3

**Published:** 2025-06-04

**Authors:** Tone Druglitrø, Silje Rebecca Morsman, Kristin Asdal

**Affiliations:** 1 TIK Centre for Technology, Innovation and Culture, University of Oslo, PB 1108 Blindern, Oslo, 0317 Norway; 2 Scandinavian University Press, Oslo, Norway

**Keywords:** Co-modification, Choreography, Atlantic cod, Immunology, Challenge experiments, Economy

## Abstract

How to make sense of the struggle of scientists in their efforts to answer demands to contribute simultaneously to the advancement of science *and* the economy? The life sciences are understood to be particularly affected by the increased institutional and political expectations to engender scientific innovations and value creation (Fochler et al., 2016: 175–200). The expectations are often closely linked to the tools that life scientists work with, such as new sequencing technologies or model organisms that are invested with hopes of novelty. The experimental life of the Atlantic cod, which is our object of study, serves here as an entry point for understanding this significant feature of contemporary life sciences. The paper shows how equipping a species to do experimental work is not necessarily about having it perform only *one type of job* Clarke & Fujimura (1992) or performing in *one*,* and exclusively one*,* site*. On the contrary, an experimental organism may be promising and interesting due to how it can be put to work to perform both in and for science, and in and for the economy, simultaneously. In analyzing the double entendre of experimental work, this paper draws upon the analytical concepts *co-modification* and *choreography* that have been carefully crafted in close empirical studies. The notion co-modification is put to work together with the notion of choreography to delineate both the material and semiotic work that go into the drawing together of the inside and outside of the lab and the material arrangements that shape the rhythm of a disciplined and controlled lab site. Together we refer to this as *choreographies of co-modification.*

## Introduction

In their introduction to the anthology, *The Right Tools for the Job: At Work in Twentieth-Century Life Sciences*, Adele Clarke and Joan Fujimura (1992) write that there is a “need for situated analyses of what is ‘guiding’ scientific work in any particular setting at any particular historical moment” (p. 4). Since then, a significant number of scholars have taken up this challenge and studied how infrastructural arrangements, instruments, and tools are made use of and shape the sciences. Studies of model organisms are one key example of this body of work (see Ankeny, 2001, 2007; Ankeny & Leonelli, 2011; Bolman, 2022b; Clause, 1993; Creager, 2002; Dam & Svendsen, 2018; Davies, 2012; Fox Keller, 2000; Gilbert, 2009; Holmberg & Ideland, 2009; Holmes, 1993; Kirk, 2008; Kohler, 1993, 1994; Logan, 2001, 2002; Lynch, 1988; Message & Greenhough, 2019; Morrison & Morgan, 1999; Nelson, 2018; Pihl, 2016; Rader, 2004; Druglitrø & Asdal, 2024; Asdal, 2014a, b; Druglitrø & Kirk, 2014). An extensive and important body of literature in the history of biology and in science and technology studies (STS), and later human-animal studies, has demonstrated how focusing on the experimental organism in science has brought new ways of understanding scientific work and its relations to culture and politics. More recently, this focus has turned into an interest in studies of the “capitalist networks” (Bolman, 2022a) formed around experimental animals (see also Ahuja, 2016; Bolman, 2022b; Davies et al., 2024; Haraway, 1989, 1997; Liao, 2024; Pihl, 2024; Rader, 2004; Suri, 2022). Taken together, such studies which focus upon the organisms in science and the investments that it takes to make them work, bring insight into how organisms are at the same time both tools and infrastructures for the study of life and the living. Moreover, they are objects upon which careers and life in research are built, while also supporting economic life outside the academy. This double entendre of work in the life sciences raises a series of concerns, issues and problematics: What are these links between the inside and the outside of the lab, and how are they forged and handled within the life sciences? How are demands and expectations to contribute to economic life while simultaneously pursuing research questions of “one’s own” shaping experimental life?

In this paper, we study these questions through the case of a non-model organism – a “non-traditional model” (Ankeny & Leonelli, 2011) – namely the Atlantic cod and how this is worked upon to do experimental work in comparative immunology and configured as an object that can lead to innovations in immunology as a field while also having socio-economic relevance in terms of a sustainable aquaculture industry. Playing on the words of Clarke and Fujimura (1992) we observe how the cod is equipped to do experimental work that is not about performing only *one type of job* or performing in *one*,* and exclusively one*,* site*,* but several.* Following on from this we seek to understand how the cod and the tasks it is equipped to perform are entwined with both epistemic and economic values.

Our analysis draws upon long-term ethnographic fieldwork among immunologists and biologists studying the immune system of the *Atlantic Cod (Gadus morhua)* as part of a project on the comparative immunology of fish and humans.In this particular paper, we follow a specific experiment known as a “challenge experiment”. Challenge experiments is an established method for evaluating the efficiency of vaccines and vaccine-induced protection in humans and farmed animals (see Gudding & van Muiswinkel, 2013; Gudding, 2010; van Muiswinckel, 2008).^1^ In the context of aquaculture, challenge experiments are regularly used exactly for these purposes and have been central to building an aquaculture industry in the first place. The first vaccine was licensed in 1976 for the prevention of yersiniosis in salmonoid fish. Since then, fish farming and fish immunology combined has become a territory of extensive scientific activity, where different species of fish have been explored in intimate ways – salmonoid species more so than any other fish. Cod has for decades been integrated in what Bolman calls “capitalist networks”, or “economized” as (Asdal & Huse, 2023) puts it, by fisheries.^2^ While salmon farming has grown into a large enterprise (Lien, 2015), the cod story is different; Despite intense efforts, cod *farming* has remained difficult. The cod tend to escape the fish pens, fail to reproduce, die, or fall ill from different infections when reared and housed on a fish farm (Asdal & Huse, 2023). The ambition of building an economically profitable and sustainable cod aquaculture industry has, however, not died out. Fish farming, including cod farming, is integral to political ambitions of building an economy for the future. Here, managing and cultivating the ocean and the organisms in the ocean are key practices where science and technology play a central role (Asdal & Huse, 2023). Funding schemes, buildings, and other infrastructures for life science research have been developed at several of the largest research institutions in Norway in the last decade to respond to policy ambitions, and the orientation towards sustainability and a future ocean economy is here key (see Treimo, 2007; Schwach, 2012; Schwach, 2000).

Our analysis of the experimental life of the Atlantic cod aims at contributing to the understanding of, first, a significant feature of contemporary life sciences, namely how scientists struggle to and are asked to contribute to the advancement of science *and* the economy (see Sigl et al., 2023; Sigl, 2019; Fochler et al., 2016), and second, how nonhuman organisms are modified and instrumentized (and thus transformed) in these efforts. In the ensuing section, we lay out the conceptual toolbox that we draw upon to tease out what we refer to as the nitty-gritty “choreographies of co-modification” that are required to plan, arrange, and execute experiments on an experimental animal like the cod, and how the cod in the same turn is “instrumentized” for immunology and the economy.

### Choreographies of co-modification: A conceptual toolbox

Conducting ethnographic research of scientific practices and working life in the life sciences over time has provided us with insight into the experimental practices and the ways in which researchers come to work with, care for, and think about the Atlantic cod as an experimental organism and what it can do. These relations, we have found, have been closely tied up to how broader contexts and demands of the life sciences are problematized and knitted into the choice of organism and the methods of the researchers. Such relationships were already pointed out by Clarke and Fujimura (1992) in *The Right Tools for the Job* but has also been problematized in more recent studies in STS that show how researchers in contemporary life sciences navigate an increasingly normative and value-laden research context (see Falkenberg et al., 2024). These studies draw mainly upon interview data and focus on individual researcher career opportunities (Fochler et al., 2016) and the reorientation of disciplines in relation to ideas of societal relevance (Sigl et al., 2023; Falkenberg et al., 2024). While working in dialogue with the above literature, our study draws upon ethnographic fieldwork and, more particularly, fieldwork that focuses on the close relationship between the experimental work of the researchers and the valuation arrangements of science. For this purpose, we have developed a conceptual toolbox that will assist us and direct our ways of ordering and systematize our observations of these relations.

“Choreography” is a metaphor and analytical concept that has been mobilized in STS studies of clinical sites, experimental practices, or practices related to care (Cussins, 1996; Law, 2010; Suzuki, 2021). Cussins (1996) introduces the concept in her ethnographic study of the clinical practices around reproductive assistance and the making of parents. “Choreography”, Cussins writes, directs attention to the “[…] materiality, structural constraint, performativity, discipline, co-dependence of setting and performers and movement” (p. 604). Cussins’ use of the notion of choreography is linked to her interest in the relationship between technological interventions and patient subjectivities (or “personhood”) in medical treatments. Law (2010) uses the notion to capture another practice, more like ours, that is about keeping different contexts, or modes of ordering, together. Law’s study is of veterinary care during the foot and mouth epidemic in the UK in 2001, where veterinarians were made responsible for slaughtering cattle on so called “contiguous premises” to prevent the spread of the disease. As Law states, there are “politics and normativities” to the strategies to stop the epidemic by mass culling, but at the same time, that of managing slaughter in a good way also demands of the veterinarian to draw upon established veterinary skills – that is, veterinary care as usual. Analysing these practices as choreographies of care, Law demonstrates how situated acts of expertise are linked up to different actors, sites, and temporalities, and are unavoidably about managing values in tension.

Three shared points from Cussins’ and Law’s way of working with the concept of choreography are particularly useful for our analysis. First, how the life science domains are tied up to and disciplined by specific societal and economic contexts. Second, how these practices involve “the intricate ordering and distribution of bodies, technologies, architectures, texts, gestures and subjectivities” in an extraordinary situation (Law, 2010, p. 9) – and the intense efforts to control (spatially and temporally) the biological and the technological. Third, how this intricate (often routine-based) ordering, or choreography, does something (or tries to do something) and creates movements and translations of both the object of concern and the sites which it is tied to.

Comparably, the concept of co-modification does related work: Co-modification has many similarities to “choreography” as it is constructed to capture the non-linear and open-ended technoscientific processes of working upon biological entities and how these practices are linked up to specific sites and ambitions (and values) (Asdal & Huse, 2023, p. 7). The concept of “co-modification” is, however, also developed in a concrete empirical site close to the site of this study, namely the investigation of research and innovation strategies oriented towards building a cod aquaculture industry (Asdal, 2015) and later developed in studies of the economization of cod (Asdal & Huse, 2023). Building upon the notion of *commodification*, co-modification is developed in this work to direct attention “to the practices and work on concrete organisms, on the one hand and how things are economized or capitalized on the other” (Asdal & Huse, 2023, p. 42). In other words, the concept is constructed to describe the processes and nitty-gritty modifications of managing the relationship between cod biology and that which is “outside” of science and tied to economization.

The notion of co-modification is in our study put to work to assist us in teasing out how the cod is being co-modified for two different sites: first, the economy by way of developing vaccination strategies for the management of cod in aquaculture, and in this way enabling a response to pathological challenges that emerge as new species are subjected to economization and innovation; and second, developing a new experimental organism for immunological and biological inquiry, that can contribute to developing the scientific community’s understanding of the immune system and alternative immune system strategies also in other species. The concept of co-modification is directed towards this “double entendre” and assists us in describing and analysing how immunologists unite basic questions of life processes and at the same time contribute to concrete innovations in domains outside of science.

The relationship between our conceptual vocabulary and the analytical work that it is assisting us can be summarized as follows: co-modification is the material and semiotic work that goes into manufacturing and literally *co-modifying* the relationship between the inside and the outside of the lab. Whereas choreography is the material arrangement that establishes the rhythm of managing the biological and the technological, the normal and the pathological, in a disciplined and controlled site. Together this is what we refer to as *choreographies of co-modification.*

The ensuing empirical analysis is divided into three distinct choreographies where we show how the scientists constantly have to adjust to and tinker with the temporal and spatial aspects of the experiment and the cod, and that of managing the experimental “assembly line”, as the scientists would often call it, that is meant to at the same time represent the aqua culture setting and the experimental setting. We show how choreographies of co-modification are done, not only in hands-on practices but also at document sites (Asdal & Reinertsen, 2022; see also Druglitrø, 2022) such as research applications, publications, and experimental protocols. Integral to instrumentizing cod is how the fish is subjected to a series of inscription practices (Latour & Woolgar, 1979), so that knowledge of the cod can travel in a modified form into new research applications, publications, experiments, and experimental protocols. We show how the challenge experiment involves instrumentizing the cod to transform them into moveable objects for both science and the economy. We empirically tease out how the experiment requires the establishment of assembly lines including a division of quite mundane tasks, from carrying heavy buckets of water and fish to more skilled tasks such as inserting the pit-tag and handling individual fish. The assembly line enables a choreography of co-modification through careful tinkering and timing.

### Co-modifying cod, immunology and the economy in research applications and experimental protocols

If asking an immunologist which model organism is the right tool for doing immunological work, there is a high probability that she will point to the mouse, and perhaps, even more specifically, to a carefully constructed mouse model known as the Balb/c 6. What does the shift in focus from the mouse model to the cod mean, practically and conceptually? Let us look more closely at how our scientists choreograph the cod’s relevance and validity as a research model, and in relation to what sites, when they write project applications for funding. Here we will see how the project applications also work as key “document-sites” for choreographies of co-modification.

In their research funding applications, as well as in much of their dissemination work, the research group tells a recurring story: this involves an article published in *Nature* in 2011 that was based on the first sequencing of the entire cod genome (by Illumina sequencing reads). With the help of new sequencing technologies, the *Nature* paper could demonstrate that the Atlantic cod has an “unusual immune architecture compared to other sequenced vertebrates” (Star et al., 2011, p. 207). The unusual “architecture” of the cod immune system was linked to the absence of the Major Histocompatibility Complex class II (MHC II) and CD + 4 T cells that are essential for the function of the adaptive immune system, such as the production of antibodies. The article stated that “this indicates how the Atlantic cod immune system has evolved compensatory mechanisms in both adaptive and innate immunity in the absence of MHC II” (p. 207). The authors did not only point out the absence of key parts of a “normal” immune system, but also suggested that since the cod was surviving and reproducing, it must build upon alternative immune strategies that so far “remain unknown” (p. 208).

Among the literature this research built upon was a review paper published in *Fisheries Science* six years prior, entitled “Why is the antibody response of cod so poor? The search for a genetic explanation” (Pilstrøm et al., 2005). The first author of the 2005 article, Lars Pilstrøm, had already studied cod biology for over two decades with a particular interest in “this strange biological phenomenon, its biochemical and genetic basis”. This 2005 paper is now a standard reference for our researchers. In addition to a few other key papers, such as Magnadottir et al. (2001) and Mikkelsen et al. (2011), the Pilstrøm paper was often referred to and discussed in project group meetings as pioneering because it pointed out the intriguing aspects of the cod immune system and the lack of knowledge about its “mechanisms”: “While it is clear that cod must have other systems (for example perhaps powerful innate immune mechanisms) to protect them from microbial infections, the nature and significance of these compensatory mechanisms is unknown” (Pilstrøm et al., 2005, pp. 967–968). Furthermore, the paper identified a gap in research on the cod immune system: “it is concluded that a deficiency in cod major histocompatibility (MH) class II molecules is a prime suspect for the lack of a specific antibody response in cod, and that testing of this hypothesis must await future experimentation” (p. 961).

Together, the referenced papers establish cod as an interesting organism for immunological study, but they also emphasize how research into the cod immune system is necessary for the species to have the capacity to work as an economic object. As one of our researchers said, “[The aquaculture industry] have to think outside the box if it is going to work with cod”. For instance, they mention that the high mortality rate of cod in captivity is a problem in terms of profitability of the practice. According to our researchers, while research has been done on cod in a farming context in the field of fish immunology, the problem is that this research has not taken into account the uniqueness of the immune system of cod. Furthermore, previous research has not made use of (or had access to) the same technology as our researchers who were now able to study gene expression in individual cells taken from immune system organs (also known as droplet single cell transcriptomics).

From the research application and project group meetings, we learn that the challenge experiment is one in a series of experiments where they aim to map the cod immune system and, through this, be able to say something about how the cod’s immune system works. “The premise” to be tested in the experiment (and coming experiments), as put in a group meeting, was that “we know that the cod has protective immunity” and “we know that the cod has poor antibody responses”. In humans and most other complex organisms, antibodies are the crucial components for immune memory specificity. The question then is what is the alternative immune strategy of the cod? Or, as put by one of the researchers in a group meeting, “there *must* be memory in cod”, but: “*What* is this memory, *where* is this memory? How is it established without CD4 help?”

When describing the relevance of the experiments, the researchers point out two main outcomes: on the one hand, the immunological research on the cod immune system is believed to potentially have an impact on how scientists come to understand immunological mechanisms in humans, and particularly autoimmune diseases. Here then, we see the same ambition as when scientists use model organisms in comparative medicine: that the animal is a stand-in for the human. At the same time, the researchers are very much interested in the cod itself. This is linked to how the cod is imagined as representing alternative strategies for survival and is approached on its own terms so to speak – researchers are genuinely interested in the cod’s biology and evolution as strategies that humans eventually can learn from. For instance, our researchers would talk about the loss of the MHC class II as “unique”, “unusual”, and “intriguing”. At the same time, they would argue for the useability of the cod in immunology as the unique immune system turned the cod into “a natural knockout system”. In their experiments, our researchers were interested in understanding how the cod has evolved over time and in encounters with pathogens – that is, the “normal situation” of the cod and how it sustains as a species, combined with the ability of the cod to sustain pathological situations that were especially “pressured”. Together, these dual perspectives were understood as knowledge that could come to inform immunological work oriented towards human health challenges. This comparative work is however a more long-term ambition as it involves transforming established immunological frameworks.

Later, we also learn that the challenge experiment will be used as empirical-experimental evidence in efforts to get funding for an add-on research project to the core project on comparative immunology in fish and humans. The add-on project responds to a call by the research council on ocean research and management and is directed towards the development of what in the call is described as “rational vaccination strategies”. The experiment, however, will simultaneously allow the researchers to continue studying immune responses in cod and to expand the immunological dimension of the cod research:

Immunological research on Atlantic cod during the first growth phase of the cod aquaculture was concerned with a practical approach to vaccination, based on the assumption that the immune system of the cod would behave similarly to other fish such as Atlantic salmon. However, we now know that the absence of MHC class II distinguishes Atlantic cod from almost all other jawed vertebrates, which indicated that new approaches are needed. (Research application 2, p. 3)

Investigating the cod as a distinct species is thus argued to be of high relevance to what our researchers in their research application call “the second wave” of cod aquaculture, and to contribute to this wave in a good way. In this way they draw together practices of understanding better the alternative strategies of the cod immune system and the development of efficient techniques and protocols for managing farmed cod better. In their application, the researchers keep these interests together, while emphasizing its distinctiveness:

Industry regulations and demands for sustainability will not allow a return to the heavy use of antibiotics characteristic of the aquaculture industry in the 20th century. Thus, strategies for rational design of vaccines are needed. The unique immune system of Atlantic cod strongly indicates that vaccinology needs to be specifically developed for this species rather than relying on knowledge from other teleost model systems. (Research Application 2, p. 7)

Their research, the application continued, would “reveal novel principles of immune regulation operating in Atlantic cod in order to develop efficient vaccination strategies and other disease-management protocols for aquaculture and wildstock”. The “challenge experiment” is then considered to be a method that can take part in such strategic work, in which figuring out how to respond to pathological challenges in aquaculture in a good way is made to stand out as a key economic necessity, a societal demand, as well as a precondition for multispecies health and welfare. We observe this co-modification of the cod in the research application – a site, or document-site (Asdal & Reinertsen, 2022), that becomes central for staging cod experiments as the core method for responding to these diverse interests and values. In the process, the cod is instrumentized as a distinct scientific and economic animal.

### Co-modifying cod, immunology and the economy: the marine research facility as the right site for the job

In this section, we turn to the second site where we study the choreographies of co-modification of cod. This is the marine research facility where the challenge experiment is conducted. The marine research facility is scientifically accredited for experiments on marine organisms and is a site where research on ocean and marine organism management has been conducted for decades. On the walls in the breakroom, 60-year-old photos hang as tokens of a long history of marine research, facilitated by The Norwegian Institute for Water Research (NIVA), including large-scale experiments directed at aquaculture. This site can thus be said to mediate between science and the economy and the longstanding relationship between fish research and fish economy. In other words, it is an established site of co-modification where both what is often called basic science *and* the economy are enacted simultaneously. It is also here that the cod will be exposed to a series of challenges, and later be translated from live fish to samples to be taken to the laboratory at the university hospital. The concrete aim of the experiment is to produce samples so that they are able to analyse the immune response on a cellular and humoral level; that is, to map exactly which kinds of immune cells and antibodies respond to the introduction of the bacteria in the cod, and in what numbers. This knowledge will be used to develop experimental protocols to enhance the understanding, expression and function of the cod immune system. As the researchers wrote in the application, and which we cited above, a key problem in efforts to farm the Atlantic cod was how vaccine strategies were not species-specific, but rather, “relying on knowledge from other teleost model systems.” (Research Application 2, p. 7). The marine research facility is, in other words, the right site at which the scientists will develop “functional” knowledge of the unique immune system of the cod. This is both interesting to explore as an immunological puzzle that has the potential to advance knowledge about alternative immune strategies. But this is knowledge that can also be used in the aqua culture setting to ensure healthy fish.

The facility is thus distinctly different from for instance the site where zebra fish would be experimented on, which is a model organism and often housed on-campus, close to the laboratory (Message & Greenhough, 2019). The marine research facility is directly perched upon the fjord. This proximity is key because it allows fresh seawater to be pumped directly into the fish tanks within the building. This creates, as put by one of the researchers, a “near to normal” situation for the fish and is “as natural as it gets” compared to a “normal” laboratory setting. At the same time, the infrastructure for the cod experiments that the facility offers is organised to mimic the aqua culture setting, however in a down-scaled and more enclosed way – as the cod is kept in tanks with seawater coming in, but at a much smaller number than in an actual fish pen for farmed fish.

The cod fish that the researchers procure for the experiment are similarly not “natural”, or a wild version of the Atlantic cod, but an already-partially-instrumentized cod; the outcome of a series of targeted and pragmatic procedures of domestication and rearing that has been experimented with over time (see Asdal & Huse, 2023). The fish that the researchers use for their experiments have been bought as smolt from the national breeding program, and then housed and reared at the marine research facility. The breeding program is based upon different species of cod, which have then become a representative of “cod” at this site. The researchers, and particularly the biologists in the project group, are keen to emphasize how there are significant differences between different cod species. One example is how the biology of the cod depends upon the level of salt in the waters that they naturally live in. Some of the researchers would point out how they were learning while working with the cod – most of them had little knowledge about how cod behaved at these sites.

For instance, regarding the relationship between the bacteria and the cod, they know already that in natural (normal) conditions, the Vibrio Anguillarum bacteria (hereafter the VA bacteria) and the cod cohabit an “ideal” situation which can be said to be held together by its own choreography of collaboration and mutual dependency (between organisms). In the aquaculture context, the cod and the VA bacteria have shown to cohabit towards a bad situation that turns pathological (Gudding, 2010). In the controlled challenge, our researchers recreate a situation that is more similar to the farm site, where the pathogenic pressure would be stronger than in natural conditions. Sustainability or the “ideal situation” is not the objective of the experiment – it is rather to establish unsustainable environments for the cod. How the cod reacts to this unsustainable environment is precisely what our researchers have less knowledge about at the outset of the experiments.

While the very site of the experiment already is shaped by a choreography of co-modification, doing experiments on cod also requires distinct choreographies that follows a protocol where keeping time and the execution of good timing is key to ensure co-modification. In the following, we show how this is a process of trials and errors where the researchers constantly also compare between and tinker with the “normal” situation and the degrees of pathogen-pressured environments in a farm setting and as the experimental setting.

### The correct dose for the job: co-modifications of cod and vibrio

The cod challenge experiment can roughly be split into two phases: what the researchers sometimes refer to as ‘the actual experiment’ and, leading up to this, ‘the trial’. In the following we will be attending to the trial. Here we tease out how the researchers construct a carefully timed choreography of cod and bacteria enacted through various (seemingly mundane) practices and material arrangements. However, the trial is key to the experiment as it is here that they will develop “the correct dose” of two serotypes of the VA bacteria, the O2a and the O2b, to later challenge the cod with in ‘the actual experiment’. In the trial, they will also learn more about the cod and how it reacts to the tank and the bacteria, and if their techniques and the timeframe set by the experimental protocol will work. Investigating the trials and errors of finding the right dose for the job thus allows us to tease out how cod resists the choreographies of co-modification, creating *counter-choreographies* that forces the researchers to be flexible and to improvise (see Suzuki, 2021, for an analysis of “improvisation” in animal research in Japan).

The trial is planned as a 10-day run in which six cod tanks are infected (‘challenged’) with one of two different vibrio serotypes - the O2a and the O2b strain - in three different dilutions. Before the trial started, the researchers had spent a full day a week before carrying 132 codfish, only a few individuals at a time in heavy water-filled buckets, from the basement of the marine research facility and into the infection unit on the floor above until there were exactly 22 fish in each of the six trial tanks. Since the move, one of the researchers explains when we come to the facility again to start the trial, the fish had now had a few days to “acclimatise to their new tank”. This is important as moving is a stressful process, and stress is a strain on the immune system, and thus also possibly the results of the trial. The cod should have been able to settle by now.

The researchers have brought six plastic bottles filled with a yellowy liquid, each bottle clearly marked with its vibrio bacteria serotype (O2a or O2b) and dilution, or dose (1, 2 or 3), such as “O2a: Dilution 1” and “O2b: Dilution 3”. This is the ‘challenge culture’ that one of the other postdocs in the project has spent the last few days in the laboratory preparing. Each serotype is prepared in three doses, each dose ten times more concentrated than the former. Doing dose trials is a balancing act. As one of the researchers puts it: “We don’t want them dying too quickly for us to sample, and we don’t want to give such a low dose that there is no infection at all!”

During the experiment, samples will be harvested from both vaccinated and unvaccinated, or naïve, cod on day “0” (a few days before infection), day 1 (day after infection), and day 4. As we can gather from the above, it is important that the dose is not so high that there are no unvaccinated individuals left alive to sample on day 4. The dose should not be too low either, as there should be some infection activating the immune system components they wish to analyse. Besides, if both unvaccinated and vaccinated fish survive the experiment, they cannot properly establish if the vaccine had any significant effect. The researchers expect the mortality of the trial tanks, from lowest to highest concentration of bacteria, to be about 20%, 50%, and 70%. The *right* dose for the job, then, is one that is lethal to most of the naïve fish within 10 days, but not so lethal as to kill all fish before day 4.

The experiment takes place in the ‘infection unit’ in the basement of the facility. The room is bare and sterile, with eight grey industrial tanks neatly lined up in rows of four on each side. Today’s tasks are written on a single sheet of paper, which looks much like a cooking recipe, including ‘adding’, ‘mixing well’ and ‘sampling’:

Reduce volume in six tanks to 200 L and stop water flow. Set up air pumps for aeration. Add challenge cultures to the six tanks and mix well. Immediately after mixing, sample 50 ml water from each of the tanks. Set up dilution series for each of the water samples, plate out dilution on blood agar plates (2.5% NaCI). Restore water flow after 1 h.

Some initial preparations are required before the vibrio bacteria can be introduced into the fish tanks, and first, to stop the flow of water. Now, and normally, a set-up of pipes and drains linked to each tank ensures a continuous flow of fresh water from the fjord outside, ensuring oxygen supply, whilst filtering out toxins. In the trial, the ‘normal situation’ is a challenge as the continuous water flow means that the bacteria, or challenge culture, quickly will be filtered out through the drains. At the same time, stopping the flow of water means simultaneously stopping the flow of oxygen. Thus, an alternative material set-up is needed, one that enables the one-hour vibrio bacteria ‘bath’ and simultaneously tends to, or sufficiently mimics, the cods’ ‘natural’ or ‘normal’ environment in the tanks. To construct *the right environment* for the job the researchers are doing, they make use of two green air pumps. They place one pump on each side of the room, each pump connected to several tubes, one lowered into each of the six tanks. The pumps are turned on, and a cluster of air bubbles visibly materialise in the water. With an alternative and workable oxygen flow now in place, the water supply is shut off. The next step is to empty the tanks to 200 L. Tiny whirlpools appear in the water as it flows down the now fully open drains. The cod respond by retreating to the sides of the tanks, away from the soft whirlpool. The researchers explain that this is to create the right dilution between water and bacteria. “With *this* amount of water, we can expect *this* dilution of the bacteria”. Preparing the bacteria is time consuming and laborious, and they cannot leave the full amount of water in the tanks, one of the researchers chimes in, “unless you want to make a whole lot more culture!”

With the flow of water stopped, alternative oxygen flows in place, and water levels properly adjusted in all tanks, the bacteria can now be introduced. The bottles’ contents are poured into the tanks, beginning with tanks A and B (the tanks were categorised from A to F). It is explained that the bacteria should be poured into the tank “like you would hand brew a cup of coffee”, that is, in carefully controlled circular movements, slowly portioning the bottle’s contents into the tank. For the next hour, we stay in the infection unit, as the researchers watch over the tanks, routinely peering into each to assess how the cod is responding to the challenge culture, discussing as they observe. The one-hour bath is a carefully timed choreography of researchers, cod, and bacteria cultures, involving coordinating levels and flows of water, oxygen, and bacteria, enacted through various practices and materials arrangements involving pumps, tubes, and keeping time, and keeping a watchful trained eye.

The trial was scheduled to go on for the next ten days, and during these ten days, we accompanied the researchers to the facility on three occasions. On our first visit, one day *post*-infection, there were few visible signs of infection, except for some redness and swelling on a few individuals in one of the tanks with the highest dosage (tank F). A concern for the slow pace of deaths quickly developed. The researchers decide to postpone the third and final sampling until seven days post-infection, but even by day seven, it is remarked that “the most relevant fish” seemed to be those in tanks E and F because they were the only ones significantly affected. By day 10, only one fish out of 42 had died in tanks C and D, and four in A and B. Eight fish had died in tank E and 11 in tank F, that is, a mortality rate of 36 and 50%, much less than the expected 70%.

In the car back to the city, we return to the question of “the correct dose”. This time, the researchers explain that “the ideal dose would be to have an 80% mortality rate”, and that they “follow the literature” when it comes to what the ‘correct’ dose is, and specifically, the most recent challenge study undertaken with vibrio and cod. In the Mikkelsen et al. (2011) study, which is the study they are referring to, most cod had died by day 7, and very few after day 10. Given, however, that all the fish they put through the trial are naïve, our researchers pointed out that one could expect *100% mortality rate* since the cod is known to have poor antibody response. As one of the researchers said, “these are all naïve fish, so *they should all die*”.

The researchers are nonetheless pleased with the experiment because they could conclude that the “technical part” of the trial had gone very well, meaning the ways in which the researchers have been able to set up an “assembly line” (“the dissection and all…this you can learn”) around the cod that is doable and efficient. With regards to the fact that the fish died at a slower rate than expected, they were not convinced that they could entirely understand the mechanisms causing this. The choreographies of the trial were thus only partially successful in the researchers’ efforts to co-modify cod and vibrio in ways that could inform them on aspects of cod in a controlled setting and that of being able to anticipate and time the cod-vibrio relationship to produce the correct dose. Both aspects would provide useful immunological and biological insight for the farmed setting and the experimental setting. The cod resists the researchers in their efforts to create the right choreography. Despite their meticulous timing and precision, the dose trial ends on day 10 without any of the six doses being concluded as ‘the correct dose’. The cod die too slowly, they do not perform according to the planned choreography. The researcher might have underrated the cod’s ability to defend itself immunologically and to resist the challenges it was put up against. The cod can in other words be said to perform a counter-choreography in a dual sense: both in terms of the infection and the experimental protocol and assembly line of the researchers. This, then, requires the researchers to improvise and take part in this counter-choreography.

As the same researcher that had judged the technical part to be successful continued to say, “the biological part, however, is… not controllable in the same way. You have to control for this… this uncontrollability.” In terms of proceeding with the experiment, controlling for uncontrollability was to be pragmatic, and – as one often must in dance in order to proceed when choreographies slip – they must improvise. There is no money, time, or fish to start over. None of the six doses, however, were ‘correct’ in the sense that none of them successfully achieved an acceptable mortality rate in cod within the allotted time of 10 days. What comes to be regarded as the right dose as well as the right time frame and duration of the experiment is enacted as malleable and flexible in the process of rendering cod properly instrumentized.

### The tools and techniques of the assembly line: co-modifications by making the Cod moveable

In the challenge experiment, the researchers were interested in cod mortality rates and the temporalities of the infection. To produce these statistics and numbers, the researchers had to establish a new assembly line that was oriented towards the endpoint of the experiment: the sampling of individual fish for analysis in the university hospital immunology lab. Sampling is what all of the choreographies of co-modification practices at the marine research facility are about. Sampling is done at specific time intervals and is also a procedure where fish are considered up close, requiring hands-on management. The researchers would take blood and spleen samples from both vaccinated and naïve individuals and from both tanks, at pre-specified (yet negotiable) timepoints. In each sampling round, the scientists need samples from both vaccinated and unvaccinated individuals and from both tanks. This is where the practice of “pit-tagging” becomes essential as an identifier and to equip the cod to become movable. To facilitate individual recognition, the research group will insert a small radio transponder with a unique ID number, known as a ‘tag’, into the abdomen of each fish. The two versions of cod would be mixed in equal numbers in the two tanks still empty in the infection unit, tanks G and H. Tank G would be challenged with O2a and tank H with O2b, both in the ‘correct dose’. The vaccinated individuals had been moved to a separate tank and vaccinated against the O2a strain of vibrio a few months earlier, this also in the form of a ‘bath’. The critical difference, of course, is that vaccines include a non-viral version of the pathogen, in contrast to the challenge culture which contains the viral version of the pathogen. Since the vaccinated cod has encountered a specific strain of the bacteria already, the researchers expected these individuals to mount a quicker and stronger immune response, that is, some kind of adaptive response based on *immunological memory*.

The pit-tagging is done as part of the preparations, in combination with moving the fish into the infection unit’s tanks G and H, a few days before the fish are introduced to the bacteria. As we drive out to the marine research facility that morning, it quickly becomes clear that we have another laborious day ahead of us. “We will probably be there all day,” one of the researchers says, “but hopefully not more than that,” adding that it all “depends on the flow of our assembly line”. Everyone – including the ethnographer – has been delegated a post for the day with a set of tasks to be repeated until all fish have been moved and tagged. Upon arrival, the day starts with moving and tagging the naïve individuals. There are more naïve than vaccinated (152 vs. 90) in the basement, leaving “more room for errors”. One of the researchers is given the task of moving fish from the basement tanks into a bucket, five at a time, and carrying the bucket up to the infection unit. This task ends at the infection unit threshold. It is now impossible to enter the unit without potentially and unintentionally dragging particles into or out of the unit. At the threshold, someone else takes the bucket, carries it across the room, and carefully pours its contents into a net, held by yet another researcher, to sieve out the water. The last person on the assembly line then carefully flips the five fish from the net into the bucket of anaesthetic.

The pit-tagging procedure does not only call for efficiency, but also precision and time sensitivity. The fish must be knocked out by the anesthetic before the tag can be inserted, which takes a few minutes, yet the fish should not be exposed to the anesthetic for more than five minutes. That is, there is quite a small window of time before the treatment becomes life threatening to the fish. The procedure is laborious exactly *because* it is time sensitive and requires a careful sensitivity, of seeing and assessing each individual fish, which again, is intimately linked to the need for a certain efficiency and ‘assembly line’ for them to be done *in time.* Pit-tagging also requires skilled handling, holding the small, slippery fish in a gentle yet firm glove-covered grip whilst puncturing its skin with the needle and injecting the tag into its abdomen with careful precision. Once the tag is successfully injected, the researchers let a scanner hover over its belly, which lets out a “beep”, affirming that the tag is in place and working, displaying a tag ID number on its screen. The fish is then gently dropped into either tank G or H and the ID number is jotted down on one of four lists: two lists for each of the two tanks, one of them for vaccinated and the other for naïve. The tag, ID number, scanner, and lists all link the individual cod to its capacity of being either vaccinated or naïve, that is, enable the fish to become instrumentized. At the same time, the very procedure allows and demands of the researchers to observe and handle the fish not merely as tools but as individuals, carefully timing and assessing the effect of anesthetic on each individual body, holding and handling them in the palm of their hand, and utilizing what they sometimes would refer to as “veterinary skills”.

In the pit-tagging procedure, the fish is given a name – for instance F22 (“F” for “Fish”), and an immunological condition (naïve or vaccinated). These names follow the fish samples. At the marine research facility, the intact cod body is worked upon, cared for, challenged, and killed, and this work is tied to the site in which it is undertaken. The cod cannot enter the hospital laboratory in this form, a site where they study the cod at the cellular and humoral level with the help of different technologies and instruments. To make the cod movable between these sites, both key to the co-modification of cod, each individual is is translated into its own “library”. This occurs at yet another site, the national sequencing center. The cod library is both an abstracted version of the cod and an amplified version of the cod, and it makes it equipped to travel to new sites.

At this point the cod has become tools that can be used in future experiments that work towards the same goals – that of instrumentizing the cod as an experimental organism and an economic object. The assembly line is not a straightforward or easily controllable process, but rather messy, contingent, and experimental, just like the trial. When moving the cod across the assembly line, the scientists learn from and with the cod, pointing out the distinctiveness of cod compared to other species of fish or between cod fish in a production setting and cod fish in a natural setting. At this point it is hard to distinguish modification work aimed at the aqua culture setting or the cod as an economic object and the modification work that is aimed at the cod as an experimentally interesting object for immunological studies; in other words, it is not possible to separate science and the economy from each other as the cod library is the result of a process of co-modification. Towards the end of the experiment, in March 2020, the COVID-19 pandemic and subsequent lock-down put a stop to any ethnographic work. The final phases of the experiment were undertaken under strict rules, and by as few people as possible. The day after the experiment had ended and any remaining surviving fish had been culled, the preliminary results and analysis were communicated, excitedly, to the whole research group by e-mail: “I have unscrambled the analysis of dead fish after we scanned all the fish yesterday. The effect of the vaccine is exactly as predicted (and better than I had dared hope for).” The preliminary analysis was a curve showing the survival rates of vaccinated versus naïve fish in each of the two tanks over the course of the experiment, known as a Kaplan-Meyer plot. The curve showed that the vaccinated fish had clearly fared better than the naïve in terms of survival rates. The excitement, however, was due to the survival rate distinction *between* the vaccinated individuals. They concluded that, “exactly as predicted”, the vaccine *did* have an effect, meaning that there must be *some sort of immunological memory* in cod. Furthermore, the results were better than they had “dared hoped for”, as it documented something which was not a given in the cod, due to what its immune system “lacks”: that there was indeed *specificity* in cod immune responses. Not only could its immune system remember previous encounters with vibrio – that is, have *immunological memory –* but this memory was quite *specific*, able to tell strains of the same bacterium apart. “We believe that vibrio anguillarum vaccination and infection model is ideal for the study of the immune response in cod”, the researchers would report to the Norwegian Food Safety Authorities (NFSA) in their retrospective assessment of the experiment.^3^ From this challenge experiment, more challenge experiments would follow that were slightly different in their technical arrangements (peritoneal injections and serum transfers). The relevance of the challenge experiments was demonstrated by the mutual construction of experimental protocols for studying immune responses in cod (*what is the alternative immune strategy of the cod?)* and the development of rational vaccination strategies that could be used in aqua culture and wild cod management.

## Conclusion: an instrumentized cod?

In this paper, we have teased out the choreographies of co-modification that are involved in innovating upon and working with the Atlantic cod in the context of immunology. Like the model organism literature has shown in detail, we have demonstrated how modifying experimental organisms to do specific jobs and to do so in a right (or good) way, is not a straightforward or given process (see Rader, 2004 in particular). What we find when following researchers working on the cod is intricate modifying work that is not only about modifying the organisms to have the capacity to do *one* job, but rather about equipping the cod in ways that make it stand out as a distinct economic and scientific animal, simultaneously. Experimental organisms are in other words key objects for “capitalist networks”, not only in terms of being commodities to be produced and distributed in a network of different economic actors, as Bolman and others have emphasized, but as we have shown here, to build new economic practices. We have shown how scientists tend to combine the relevance to economic practices and working on problems that they themselves (and their field) finds to be interesting, important and relevant and that might be linked to more fundamental questions about life and life processes. The cod demands of the researchers to “start over”, to ask other questions, make use of new technologies, and thus also arrange experiments in new ways.

As cod is experimented upon cod is modified for something; it is carefully equipped, *instrumentized* or *apparatized* with the capacity to do work in and across sites of science and the economy. *Co-modification*, we have shown, is useful to tease out these nitty-gritty practices. In turn, it is helpful to begin to demonstrate how the stretch that researchers in the life sciences find themselves to be disciplined by and contingent upon – that of the intense demands related to being in and working in academia (as Fochler et al., 2016 has called “hypercompetition”), the legal, scientific, and ethical demands related to working on research organisms, and the demands of doing research of high relevance and productivity to society and the economy – is enacted in concrete work practices and in concrete settings. The research on cod answers to financial structures that ask researchers to engage in innovative and interdisciplinary research. At the same time, researchers in the life sciences are asked to be explicit about their contributions to larger societal (and economic) challenges, such as the need to develop industries for the future. More immediate and tangible outcomes thus need to be linked to research practices. It is here that the double entendre of cod becomes relevant: the research that we have been following is both about innovations in immunology and about modifying the cod as a distinct economic object.

## Footnotes

1 The method can be linked back to Edward Jenner’s experiments on inoculation in the late 1700s, even though Jenner never used the notion of “challenge experiments” or “challenge trials”. The basic premise however – that of intentionally challenging an organism with an infectious disease organism to study how immunological memory is formed – was also integral to Jenner’s method. The use of fish in research in Norway today is almost exclusively linked to vaccine development and/or the aquaculture industry (over 2 million fish were registered for use in scientific experiments in 2021, see Norwegian Food Safety Agency, 2022; see also Research Council of Norway, 2009).

2 See Marianne Lien’s ethnographic study Becoming Salmon: Aquaculture and the Domestication of a Fish (2015) on the salmon aquaculture industry and the growth of domestication practices in marine environments. See also Yu Kai-Liao’s (2024) study of shrimps in labs and how biosecurity measures developed in the lab relate to capitalist forms of life and relations.

3 Retrospective Assessment submitted to the NFSA, 2021.
