# Correction: Choreographies of co-modification: instrumentizing cod for immunology and the economy

**DOI:** 10.1007/s40656-025-00685-3

**Published:** 2025-07-16

**Authors:** Tone Druglitrø, Silje Rebecca Morsman, Kristin Asdal

**Affiliations:** 1 TIK Centre for Technology, Innovation and Culture, University of Oslo, Blindern, Oslo, 1108, 0317 PB Norway; 2 Scandinavian University Press, Oslo, Norway

**History and Philosophy of the Life Sciences (2025) 47:29**

10.1007/s40656-025-00677-3

In this article the reference to 'Fochler et al., (Minerva, 54:175–200, 2016)' in the abstract, should be '(Fochler et al, 2016: 175-200)'; the reference to 'AUTHOR' in the paragraph starting with 'The cod fish that the researchers procure' should instead have referred to 'Asdal & Huse’.

The original article has been corrected.

